# History of Cesarean Section Associated with Childhood Onset of T1DM in Newfoundland and Labrador, Canada

**DOI:** 10.1155/2012/635097

**Published:** 2012-07-08

**Authors:** J. Phillips, N. Gill, K. Sikdar, S. Penney, L. A. Newhook

**Affiliations:** ^1^Newfoundland and Labrador Centre for Health Information, St. John's, NL, Canada A1B 2C7; ^2^Janeway Pediatric Research Unit, 4th Floor Janeway Hostel, Memorial University of Newfoundland, 300 Prince Philip Drive, St. John's, NL, Canada A1B 3V6; ^3^Janeway Children's Health and Rehabilitation Centre, 300 Prince Philip Drive, St. John's, NL, Canada A1B 3V6

## Abstract

*Objectives*. Newfoundland and Labrador (NL) has one of the highest incidences of Type 1 diabetes mellitus (T1DM) worldwide. Rates of T1DM are increasing and the search for environmental factors that may be contributing to this increase is continuing. *Methods*. This was a population-based case control design involving the linkage of data from a diabetes database with live birth registration data. 266 children aged 0–15 years with T1DM were compared to age- and gender-matched controls. Chi-square analysis and multivariate conditional logistic regression were carried out to assess maternal and infant factors (including maternal age, marital status, education, T1DM, hypertension, birth order, delivery method, gestational age, size-for-gestational-age, and birth weight). *Results*. Cases of T1DM were more likely to be large-for-gestational-age (*P* = 0.024) and delivered by C-section (*P* = 0.009) as compared to controls. C-section delivery was associated with increased risk of T1DM (HR 1.41, *P* = 0.015) when birth weight and gestational age were included in the model, but not when size-for-gestational-age was included (HR 1.3, *P* = 0.076). *Conclusions*. Birth by C-section was found to be a risk factor for the development of T1DM in a region with high rates of T1DM and birth by C-section. These findings may have an impact on health practice, health care planning, and future research.

## 1. Introduction

Type 1 diabetes mellitus (T1DM) is one of the most common chronic diseases in childhood and results from autoimmune destruction of pancreatic *β*-cells, leading to insulin deficiency. T1DM is thought to originate through a combination of genetic and unknown environmental factors, of which environmental factors remain poorly defined. Newfoundland and Labrador (NL), Canada, is recognized as having one of the highest rates of T1DM worldwide [1]. A study on hospitalizations of children in NL reported an increase in diabetes-related hospitalizations among children aged 0–19 years [2]. T1DM is a significant disease in NL with its associated acute and chronic complications as well as the economic costs to both families and the health care system. Identification of potential perinatal environmental risk factors is examined in this study to try and elucidate potential reasons of why the disease is so common in this region of North America. 

## 2. Methodology

This study was a case control design involving the linkage of data extracted from the Newfoundland and Labrador Diabetes Database (NLDD) with the Live Birth System (LBS). The NLDD is maintained by the Janeway Pediatric Research Unit at the Janeway Child Health Care Centre (JCHCC) in St. John's, NL. The JCHCC is the only tertiary care pediatric hospital in the province. The NLDD includes data for the majority of cases of T1DM diagnosed in NL from 1987 to present. Children are included in the database as part of a prospective provincial study on the epidemiology of T1DM in NL. They have a confirmed diagnosis of T1DM according to Canadian Diabetes Association (CDA) criteria [3]. The LBS is maintained by the Newfoundland and Labrador Centre for Health Information (NLCHI). Data for this system are acquired from Live Birth Notification forms that are completed in all provincial health care facilities. The forms are provided to NLCHI by the Vital Statistics Division, Service NL, and contain clinical and demographic data for all births (resident and nonresident) in the province. The system currently contains data on all births from 1992 to 2011.

Patient records were individually linked across datasets. Cases included children born in NL from 1992 onwards which have been diagnosed with T1DM before 15 years of age. Children with type 2 diabetes, maturity-onset diabetes of youth, transient hyperglycemia, and diabetes caused by chemotherapy or cystic fibrosis are excluded from the NLDD and thus were not included in the study. Children born prior to 1992 were not included in the study because there are no electronic birth notification data available before this date. 

A unique identifier, such as the provincial health insurance plan number, was not available for all children in the NLDD. As a result case subjects were linked to the LBS using child's date of birth and mother's maiden name. Where available, child's name was used to verify the linkage. Of the 301 cases in the NLDD, 23 were excluded because they were born out of province. Of the remaining 278, 6 were excluded as duplicate records. Linkage was possible for all but six children resulting in a total of 266 cases included in the study. Three control subjects (*N* = 798) were selected for each case matched on year of birth, sex, and health authority of mother's residence at time of delivery. Power analysis was performed to determine whether this sample size would be sufficient to detect statistical significant associations between T1DM and the risk factors of interest. The power analysis was conducted considering an overall rate of birth by C-section in NL as 30.9% [4], in order to achieve a power of 80% with a desired odds ratio of 1.5. Using the method described by Kelsey et al. [5] the power analysis confirmed that a sample size of 266 cases and 798 controls is sufficient to detect statistically significant relationships between T1DM and delivery by C-section.

Cases and controls were grouped into two gestational age categories: preterm and term/postterm. Birth weight in grams was used to classify cases and controls as high birth weight (>4,000 grams) or not (≤4,000 grams). Cases and controls were also classified as small/appropriate-for-gestational-age or large-for-gestational-age using the method described by Kramer et al. [6]. Method of delivery was categorized as vaginal or C-section. Cases and controls were grouped according to parity or birth order as either 1 and 2 or more. Mother's age in years was classified as ≤34 years or >34 years. Mothers were also classified by their T1DM status and hypertension status. Mother's marital status was categorized as married, single, separated, widowed, or divorced. Mother's education level was classified into three categories: not graduated high school, graduated high school, and education beyond high school.

Descriptive statistics were generated to describe the distribution of cases and controls. Demographic and clinical factors of mothers, including age, marital status, education, place of residence, parity (number of live born children delivered), and complications of pregnancy, were included. Cases and controls were analyzed by sex, place of residence, age of onset, length of gestation, type of delivery, birth weight, size for gestational age, and birth order. 

Chi-square tests were used to predict diabetes status on the basis of the independent variables. Conditional multiple logistic regression was used to assess the relationship between T1DM risk and the variables of interest. Two conditional logistic regression models were employed. The first model contained birth weight, gestational age, parity, delivery method, mother's marital status, mother's education level, mother's age, maternal hypertension, and mother's T1DM status. The second model incorporated all variables in the first model with the exception of birth weight and gestational age which were replaced with size-for-gestational-age. Birth weight and gestational age were not included in the same model as size-for-gestational-age as they are components of this variable. 

The Statistical Package for the Social Sciences (SPSS) 17.0 was used to generate descriptive statistics and chi-squares. SAS 9.2 was used to conduct the conditional multivariate logistic regressions.

This study received approvals from the Human Investigation Committee of Memorial University, from each of the hospital boards and the Secondary Uses Committee of the Newfoundland and Labrador Centre for Health Information (NLCHI) prior to commencement.

## 3. Results

Table 1 presents the descriptive characteristics of the cases of T1DM. The percentages of male and female cases were similar (50.8 and 49.2, resp.). Table 1 also demonstrates the age distribution of cases as well as their age of diagnosis. There were more males than females diagnosed in the 0–4 age group; however, this finding was not statistically significant.

Table 2 presents maternal and perinatal characteristics of the study population. A higher percentage of cases than controls were born pre-term (9.8% versus 6.8%, resp.). While there was no significant difference observed between birth weight of the cases and controls, there was a significant difference observed for size-for-gestational-age with a higher percentage of cases than controls born large-for-gestational-age (18.2% versus 12.8%, resp., *P* = 0.024). It was more common for cases to be delivered by C-section than controls (30.8% versus 22.1%, *P* = 0.009). T1DM was more common among first or second born cases compared to those born third or higher (*P* = 0.022).

Table 3 presents the results of the conditional logistic regression models. In the model which included birth weight and gestational age, delivery by C-section was associated with increased risk of T1DM. Children delivered by C-section were 1.41 times as likely to develop T1DM (Hazard ratio (HR) 1.41, *P* = 0.015). In the second model, which included size-for-gestational-age, C-section delivery was not associated with increased risk of T1DM (HR 1.3, *P* = 0.076). Both parity and size-for-gestational-age were found to be significant risk factors for T1DM from chi-square analysis (Table 2); these factors did not remain statistically significant in the conditional logistic regression models.

## 4. Discussion

Findings of this study indicate that C-section delivery was a significant risk factor for T1DM in children aged 0–15 years. This finding is in line with a recent meta-analysis of 20 studies which found that the combined effect of C-section delivery was 1.23 (95% CI 1.15–1.32) [7]. Theories of why this may be associated with the development of T1DM in offspring includes the involvement of the role of gut bacteria in the development of the immune system [8]. Studies have shown a difference between the compositions of gut microbiota in vaginally delivered children and those delivered by C-section. Children delivered by C-section may be primarily exposed to bacteria in the hospital and not maternal bacteria, hence the increased risk of T1DM may be linked to a different composition of gut flora [8]. Another possible explanation is related to the hygiene hypothesis which proposes that the risk of diabetes may be increased when children are not exposed to infections in early life [9]. Children delivered by C-section have decreased exposures to infections compared to children born vaginally and, in turn, have increased risk for diabetes [9]. Another theory suggests that the observed increased risk of diabetes after C-section may be related to perinatal stress [10]. NL has a high rate of birth by C-section as compared to other regions in Canada. The provincial rate of births by C-section was 30.9% in 2005-2006 versus the Canadian rate of 26.3% [4]. The rates of C-section have increased in NL to 33% in 2010 [11].

In the present study, maternal age at time of birth was not found to be significantly associated with risk of T1DM in offspring; other studies have found significant relationships between mother's age and T1DM risk. A recent meta-analysis of 37 studies found that the odds of T1DM increased by 10% for children whose mothers were over 35 years of age at time of birth (OR = 1.10 95% CI 1.01, 1.20; *P* = 0.03) [12]. Conversely, a matched case-control study of 196 cases in the United Kingdom [13] found that mothers of control children were older than mothers of cases (OR = 0.900 95% CI 0.854, 0.948; *P* < 0.001). For other maternal factors, such as education level and marital status, there were no associations found which is consistent with other similar studies [14, 15]. The present study also did not find any associations between maternal hypertension and risk of T1DM in offspring. Other studies have found an increased risk of T1DM in offspring with maternal history of T1DM [13, 16, 17]; however, these studies also included information on paternal history of T1DM. A 2009 study by Algert and colleagues [18] did not find an association between maternal T1DM and risk of T1DM in children. Similar to the present study, the study by Algert et al. did not contain information on paternal T1DM.

There was no significant relationship between parity and T1DM risk found in the current study. This is different than a Western Australia population based cohort study of 835 cases of T1DM diagnosed by the age of 15 that found a significant decrease in T1DM with increasing birth order [18]. 

Birth weight and gestational age were not found to be associated with risk of T1DM in the present study; however, chi-squared analysis revealed a significant difference between T1DM and size-for-gestational-age with a higher percentage of cases than controls born large-for-gestational-age, but this was no longer significant in the conditional logistic regression models. Our findings do not support the findings of a meta-analysis of 11 studies examining birth weight which found that a birth weight greater than 4,000 grams was associated with an increased odds of T1DM (OR = 1.17 95% CI 1.09, 1.26; *P* < 0.05) [19]. Findings related to the association between gestational age and T1DM appear to be mixed. A case-control study conducted in Austria found that babies born at 34–39 weeks had a significantly higher risk for T1DM compared to those born before 33 or after 40 weeks [20]. However, a study by Cardwell et al. [21] found that children born after 40 weeks gestation had a significantly lower risk of T1DM than children born prior to 40 weeks. While size-for-gestational-age has not been extensively studied, some studies have found significant associations. A cohort study of 272 children in New South Wales, Australia, with T1DM found that children who were small-for-gestational-age had a significantly decreased risk of T1DM compared to children born appropriate-for-gestational age [18].

The findings of this study should be considered in the context of its strengths and weaknesses. An important strength is that the use of a record linkage case-control study design eliminates recall bias that is apparent in cross-sectional study designs. Secondly, data contained in the LBS were collected at time of birth by healthcare professionals, and the NLDD data were collected from physician charts at time of T1DM diagnosis which contributes to the reliability of the data. A limitation of this study is that there was very little information available pertaining to fathers as the majority of the information collected at the time of birth for the LBS is related to the mother and child. Thus, paternal factors and family history could not be considered for analysis. 

This study identified C-section as a significant risk factor for the development of T1DM among children aged 0–15 years in NL, a region with very high rates of T1DM. Findings may have an impact on health practice, health care planning and future research related to T1DM among children. Further research should be undertaken to understand the nature of this association.

## Figures and Tables

**Table 1:** Characteristics of children diagnosed with T1DM in Newfoundland and Labrador, by sex, 1992–2010.

							
Variables	Males		Females		Total		*P* value
	*n*	%	*n*	%	*n*	%	
Year of birth						
1992–1995	59	43.7	54	41.2	113	42.5	0.553
1996–1999	34	25.2	43	32.8	77	28.9	
2000–2003	25	18.5	21	16.0	46	17.3	
2004–2007	17	12.6	13	9.9	30	11.3	
Year of diagnosis							
1993–1997	12	9.1	6	4.7	18	6.9	0.263
1998–2002	39	29.5	32	25.0	72	27.6	
2003–2006	33	25.0	31	24.2	64	24.1	
2007–2010	48	36.4	59	46.1	107	41.0	
Age at diagnosis							
0–4	55	41.7	36	21.8	91	35.0	
5–9	48	36.4	57	44.5	105	40.4	0.073
10–15	29	22.0	35	27.3	64	24.6	

**Table 2:** Maternal and perinatal characteristics of the study population.

					
Variables	No. of cases (*N* = 266)		No. of controls (*N* = 798)		*P* value
	*n*	%	*n*	%	
Gestational age (completed weeks)				
Preterm	26	9.8	54	6.8	
Term/postterm	240	90.2	743	93.2	0.073
Birth weight (grams)					
<2,500	16	6.0	43	5.4	
2,500–4,000	204	76.7	622	78.1	
>4,000	46	17.3	131	16.5	0.873
Size-for-gestational-age					
Small/appropriate	207	81.8	679	87.2	
Large-for-gestational-age	46	18.2	100	12.8	0.024^∗^
Method of delivery					
Vaginal spontaneous	147	55.3	519	65.0	
Vaginal assisted	37	13.9	103	12.9	
C-section	82	30.8	176	22.1	0.009^∗^
Mother's age (years)					
≤34	235	88.3	725	90.9	
>34	31	11.7	73	9.1	0.142
Mother has T1DM					
Yes	4	1.5	9	1.1	
No	262	98.5	789	98.9	0.416
Mother has hypertension					
Yes	18	6.8	56	7.0	
No	248	93.2	742	93.0	0.508
Mother's marital status					
Married	181	68.0	499	62.5	
Single, separated, widowed, divorced	85	32.0	299	37.5	0.060
Birth order (including current live birth)					
1-2	234	88.0	653	48.4	
3+	32	12.0	145	18.2	0.022^∗^
Education					
Not graduated high school	39	15.1	138	17.7	
Graduated high school	49	18.9	166	21.3	
Education beyond high school	171	66.0	475	61.0	0.344

*P value of less than 0.05 was considered significant.

**Table 3:** Risk of T1DM associated with specified maternal and perinatal factors.

Variables	Birth weight/gestational age model		Size-for-gestational-age model	
	HR	*P* value	HR	*P* value
Birth weight				
≤4,000	REF		—	—
>4,000	1.07	0.692	—	—
Gestational age				
Preterm	REF		—	—
Term	0.777	0.282	—	—
Size-for-gestational age				
Small/appropriate	—	—	REF	
Large	—	—	1.33	0.112
Delivery method				
Vaginal	REF		REF	
C-section	1.41	0.015	1.304	0.076
Mother's age (years)				
≤34	REF		REF	
>34	1.14	0.531	1.15	0.5199
Mother has T1DM				
No	REF		REF	
Yes	1.16	0.795	1.129	0.388
Mother has hypertension				
No	REF		REF	
Yes	0.836	0.502	0.930	0.552
Mother's marital status				
Married	REF		REF	
Single, separated, widowed, divorced	0.880	0.654	0.849	0.3003
Parity				
1	REF		REF	
2+	1.04	0.787	1.010	0.942
Mother's education				
Has not graduated high school	0.880	0.520	0.898	0.597
Graduated high school	0.889	0.506	0.907	0.592
Education beyond high school	REF		REF	

T1DM: type 1 diabetes mellitus.
